# Supplementary material for: Expression and molecular characterization of an intriguing hyaluronan synthase (HAS) from the symbiont “Candidatus Mycoplasma liparidae” in snailfish
Source: PeerJ. 2025 Apr 25;13:e19253. doi: 10.7717/peerj.19253 (PMC12036578; doi:10.7717/peerj.19253)
Supplement: Supplemental Information 2 [file peerj-13-19253-s002.docx]

| Gene | Forward Primer (5’-3’) | Reverse Primer (5’-3’) |
| --- | --- | --- |
| snHAS | ATGAAATATACTATTCTTATTCC | TATATAGAATATATGGATATTAA |
| the mutant primer | TCTTTTTGTCAGGA**C**TATAATTATTTTGAAGATGCTGG | CCAGCATCTTCAAAATAATTATAGTCCTGACAAAAAGA |
