# Supplementary material for: Expression and molecular characterization of an intriguing hyaluronan synthase (HAS) from the symbiont “Candidatus Mycoplasma liparidae” in snailfish
Source: PeerJ. 2025 Apr 25;13:e19253. doi: 10.7717/peerj.19253 (PMC12036578; doi:10.7717/peerj.19253)
Supplement: Supplemental Information 3 [file peerj-13-19253-s003.docx]

| **Organisms** | **GenBank NO.** | **Class** | **Transmembrane regions** | **Amino acid number** | **MW(molecular weight, kDa)** |
| --- | --- | --- | --- | --- | --- |
| Candidatus Mycoplasma | **WWS21520** | - | **0** | **310** | **37.2** |
| Mycoplasma | MDK2819671 |  | 0 | 310 | 37.26 |
|  | MDR2568307 |  | 1 | 355 | 42.6 |
|  | MDR2847095 |  | 1 | 348 | 41.39 |
| Xenopus laevis | XP_018123734 | Class I | 6 | 552 | 63.83 |
| Heterocephalus glaber | JAN99696 |  | 6 | 552 | 63.47 |
| Homo sapiens | NP_001514 |  | 7 | 578 | 64.84 |
| Streptococcus equi | ACG61577 |  | 5 | 417 | 44.78 |
| Streptococcus dysgalactiae | QDJ95207 |  | 5 | 439 | 50.35 |
| Streptococcus uberis | AUC25715 |  | 5 | 417 | 47.33 |
| Streptococcus pyogenes | AAK34828 |  | 5 | 419 | 47.92 |
| Chlorella virus | NP_048446.1 |  | 7 | 568 | 65.17 |
| Pasturella multocida | AAC38318 | Class II | 2 | 972 | 112 |
| *Neobacillus niacini* | WP_310177728.1 | other | 0 | 322 | 37.38 |
| Thermoclostridium stercorarium | WP_015357958.1 |  | 0 | 290 | 33.72 |
| *Melainabacteria* bacterium | RAI11187.1 |  | 0 | 285 | 32.82 |
| *Mediterraneibacter glycyrrhizinilyticus* | WP_205154686.1 |  | 0 | 304 | 36.02 |
| *Lactococcus lactis* | WP_289446540.1 |  | 0 | 320 | 36.95 |
| *Fusobacterium mortiferum* | WP_005886709.1 |  | 0 | 339 | 40.72 |
| Mycoplasmataceae bacterium | MDR1235299.1 |  | 0 | 330 | 39.73 |
| *Parageobacillus thermoglucosidasius* | WP_003247850.1 |  | 0 | 324 | 38.22 |
| *Thomasclavelia spiroformis* | WP_278623572.1 | other | 0 | 308 | 35.91 |
| *Heyndrickxia coagulans* | WP_110133786.1 |  | 0 | 318 | 37.00 |
| *Turicibacter bilis* | WP_212723917.1 |  | 0 | 330 | 38.97 |
| *Fusobacterium mortiferum* | RGM96983.1 |  | 0 | 350 | 41.76 |
| *Leuconostoc fallax* | WP_170168005.1 |  | 0 | 328 | 39.36 |
| *Rhodococcus qingshengii* | TDL61521.1 |  | 0 | 348 | 41.02 |
| Erysipelotrichaceae bacterium | MCI5701658.1 |  | 0 | 315 | 37.51 |
| *Methanobrevibacter woesei* | WP_273476813.1 |  | 0 | 379 | 44.40 |
| *Clostridium hominis* | WP_207725341.1 |  | 0 | 350 | 42.03 |
| *Epilithonimonas vandammei* | WP_313598802.1 |  | 0 | 350 | 40.99 |
| *Fusobacterium mortiferum* | MBM6691184.1 |  | 0 | 356 | 42.94 |
