# Supplementary material for: Expression and molecular characterization of an intriguing hyaluronan synthase (HAS) from the symbiont “Candidatus Mycoplasma liparidae” in snailfish
Source: PeerJ. 2025 Apr 25;13:e19253. doi: 10.7717/peerj.19253 (PMC12036578; doi:10.7717/peerj.19253)
Supplement: Supplemental Information 4 [file peerj-13-19253-s004.docx]

| **HA Standards (kDa)** | **Retention time (min)** |
| --- | --- |
| 708 | 31.17 |
| 344 | 32.35 |
| 200 | 34.02 |
| 107 | 35.81 |
| 47.1 | 37.70 |
| 21.1 | 39.45 |
| 9.6 | 41.50 |
| 5.9 | 42.60 |
